# Molecular Biocompatibility Assessment of PETG Aligners After Processing by Laser or Milling

**DOI:** 10.3390/ma18204793

**Published:** 2025-10-20

**Authors:** Katia Barbaro, Ginevra Ciurli, Ettore Candida, Francesca Silvestrini-Biavati, Valentina Lanteri, Paola Ghisellini, Cristina Rando, Roberto Eggenhöffner, Alessandro Ugolini

**Affiliations:** 1 Istituto Zooprofilattico Sperimentale del Lazio e della Toscana “M. Aleandri”, 00178 Rome, Italy; katia.barbaro@izslt.it (K.B.); ginevra.ciurli-esterno@izslt.it (G.C.); 2 Department of Surgical Sciences and Integrated Diagnostics (DISC), Genova University, 16132 Genoa, Italy; ettore.candida@gmail.com (E.C.); silvestrini.fra@gmail.com (F.S.-B.); paola.ghisellini@unige.it (P.G.); cristina.rando@unige.it (C.R.); roberto.eggenhoffner@unige.it (R.E.); 3 Surgical, Medical and Dental Department, University of Modena and Reggio Emilia, 41124 Modena, Italy; valentina.lanteri@unimore.it

**Keywords:** PETG biocompatibility, laser/milling cutting, endocrine disruptors, gene expression profiling, inflammatory response

## Abstract

Polyethylene terephthalate glycol-modified (PETG) is a transparent, stable copolymer commonly used in biomedical devices such as surgical guides, clear aligners, and anatomical models. Its biocompatibility must be assessed not only for cytotoxicity, but also for subtle molecular and immunological responses, especially when in contact with mucosal or hormone-sensitive tissues. This study evaluated the biological safety of PETG processed via CNC milling and CO_2_ laser cutting, two methods that preserve bulk chemistry but may alter surface properties. PETG diskettes were analyzed by FT-IR, ^1^H-NMR, and GC–MS to confirm chemical integrity and absence of degradation products. Biocompatibility was tested using MCF-7 epithelial cells and THP-1 monocytes. Cell viability remained above 90% over seven days. Inflammatory (COX-2, TNFα, IL-8, IL-1α, IL-4, IL-10, IFNγ) and hormone-related (ERα, ERβ) gene expression was analyzed by qRT-PCR. Gene profiling revealed only modest, non-significant changes: COX-2 was upregulated 1.8-fold after laser processing, and ERα increased 1.6-fold following milling—both below thresholds considered biologically active. These findings indicate that mechanical surface treatments induce minimal bioactivity, with no meaningful immune or hormonal stimulation. PETG remains functionally inert under the tested conditions, supporting its continued safe use in intraoral and hormone-sensitive biomedical applications.

## 1. Introduction

Polyethylene terephthalate glycol-modified (PETG) is a thermoplastic copolyester commonly utilized in the field of biomedical applications due to its transparency and its biomechanical properties [1,2]. Further, its ability to be compatible with additive and subtractive fabrication has widened application fields that span anatomical surgical guides, temporary implant scaffolds, and mechanisms for drug delivery [1]. Also, in dentistry, clear aligners, used for orthodontic treatments, can be specially made of glycol-modified polyethylene terephthalate (PETG), because its mechanical properties are suitable for the intraoral environment [3]. While PETG is generally accepted to be chemically stable and biocompatible, changes triggered during fabrication through laser cutting and CNC milling can potentially alter surface properties with possible influences on cellular responses.

Mechanical and heat treatment processes can alter the microtopography of the polymer and chemistry on its surface, and in doing so release microparticles, heat degradation products, and low-molecular-weight oligomers [4]. Although they are not overtly toxic on their own, these substances can interact with the molecular sensors of human cells, potentially inducing stress reactions, modifying inflammatory gene expression patterns, and regulating hormone receptor activity. These substances interact with the molecular sensors of human cells, potentially inducing stress reactions, modifying inflammatory gene expression patterns, and regulating stress reactions. Therefore, biocompatibility tests need to go beyond simple viability tests; it is necessary to add transcriptional assessment to more adequately and sensitively detect sub-toxic but biologically relevant changes.

Despite extensive characterization of the bulk matrix and mechanical properties of PETG, few studies have addressed bioreactivity after clinically relevant surface modifications for PETG [5]. Particularly, its property of being molecularly neutral—defined as the absence of biological activation through immune or endocrine pathways—is poorly studied. Recent clinical and laboratory studies have demonstrated that PETG is generally safe for use in orthodontics, although there have been slight indications of estrogenic activity. Research on commercial aligners found no signs of toxicity but did observe a mild increase in estrogen receptor gene expression. Despite these findings, how manufacturing techniques like milling or laser cutting affect PETG’s biological safety has not yet been investigated—a gap that this study aims to address [6].

To address this problem, we carried out a biocompatibility test on mechanically milled and laser-milled PETG diskettes [7]. We used analytical chemistry on surface-treated PETG samples in search of possible leaching compounds and to ensure that the polymer was still unaltered. Two different human cell lines for biological tests were used: THP-1, that is an immune cell line from a monocyte that responds to inflammatory stimuli, and MCF-7, i.e., a breast epithelial model that responds to environmental changes and estrogens. Among parameters tested were viability (MTT), pro-inflammatory mediator expression (COX-2, IL-6, IL-8) and estrogen receptor expression (ERα and ERβ).

The aim of this study is to determine if the surface treatment alters the chemical or biological neutrality of PETG in vitro. This combined approach provides a broad assessment of biocompatibility, as recommended in biomaterials testing.

A material that maintains transcriptional silence across inflammatory and hormonal pathways can be considered truly biocompatible, as this goes beyond the simple concept of minimal toxicity. The findings provide critical information for determining the safety of PETG for biomedical applications in which contact with hormone-responsive or sensitive tissues is likely.

## 2. Materials and Methods

### 2.1. PETG Sample Preparation

The thermoplastic material used in this study is Hybrid copolyester of PET-G [Nuvola Essential, Linea Giove, G.E.O. S.r.l. (Gruppo Europeo di Ortodonzia) Via del Progresso 14–00030 San Cesareo (RM), Italy]. It was used to create circular diskettes that were 10 mm in diameter and 1 mm thick, which were then cut on the 3D-printed model. To replicate actual clinical fabrication methods, the samples were exposed to two different surface processing techniques: CO_2_ laser cutting (Infinity Plus, Iradion Laser Inc., Uxbridge, MA, USA) and CNC milling (Tungsten Carbide Drill coated with DLC). A computer-controlled 3-axis milling system was used to machine anatomically shaped PETG blocks to produce diskettes under milling conditions. The cutting parameters were chosen to prevent surface deformation: spindle speeds of 12,000 rpm and a feed rate of 30 mm/min. Laser-treated samples with poor carbonization and clean edge definition were created using a Class I CO_2_ laser cutter set to operate at a wavelength of 10.6 µm, with a pulse width of 100 µs and an average power of 30 W. Every diskette was carefully cleaned three times in sterile phosphate-buffered saline (PBS) after production to remove any debris, and then it was left to air dry in a sterile environment with laminar flow. When handling the samples, autoclaved forceps and nitrile gloves were used to prevent contamination.

### 2.2. Chemical Characterization

A combination of spectroscopic and chromatographic techniques was employed to assess the chemical stability and potential leaching behavior of PETG [8,9]. Fourier-transform infrared spectroscopy (FT-IR) was carried out using a Bruker-Alpha FT-IR spectrometer (Bruker, Billerica, MA, USA) equipped with an attenuated total reflectance (ATR) accessory. Spectra were collected in the range of 4000–400 cm^−1^ at a resolution of 4 cm^−1^, averaged over 32 scans per sample. The presence or absence of characteristic functional groups, including aromatic rings, ester carbonyls, and hydroxyl moieties, was compared to reference spectra of virgin known PETG.

Proton nuclear magnetic resonance (^1^H-NMR) was performed to detect the presence of residual monomers or potential contaminants not identifiable by FT-IR. PETG fragments were dissolved in 5–10% *w*/*v* in CDCl3 and spectra were acquired with a Varian Mercury Plus VX 400 (1H, 399.9; 13C, 100.6 MHz) spectrometer (Varian Inc., Palo Alto, CA, USA). A spectral window of 0–10 ppm was employed, and particular attention was given to the aromatic region between 6.5 and 7.5 ppm, where signals from bisphenol A or phthalate-related compounds would typically appear [10]. The absence of peaks corresponding to known endocrine disruptors was verified by comparison with mass spectral libraries.

Gas chromatography coupled to mass spectrometry (GC-MS) was used to analyze potential leachable compounds. PETG samples were immersed in methanol (analytical grade) and dissolved in dichloromethane in presence of m-fluorotoluene as internal standard (200 ppm). The extracts were then injected into Thermo FOCUS GC system coupled with a Thermo DSQ single quadrupole mass spectrometer (Thermo Fisher Scientific, Waltham, MA, USA).

### 2.3. Cell Lines and Culture Conditions

The biological reactivity of the processed PETG diskettes was assessed using two human cell lines. The ATCC provided MCF-7 cells, a human breast adenocarcinoma line that is sensitive to estrogenic stimuli. The cells were cultured in high-glucose medium (DMEM) supplemented with 10% fetal bovine serum (FBS), 1% penicillin–streptomycin, and kept at 37 °C in a humidified 5% CO_2_ atmosphere. THP-1 cells, a human monocytic leukemia line (ATCC TIB-202), were cultivated in RPMI-1640 medium with 10% FBS and 1% antibiotics under the same incubation conditions as a model for immunological responsiveness [11].

Twenty-four hours before treatment, cells were seeded in 24-well plates at densities of 1 × 10^5^ cells per well for MCF-7 and 2 × 10^5^ cells per well for THP-1. Just before the experimental incubation periods began, PETG diskettes were gently inserted into contact with the cell monolayers or suspension. Gene expression analyses were performed at 24 and 48 h, while periods of exposure for viability assays were extended to 7 days.

### 2.4. Cell Viability Assay (MTT)

The colorimetric MTT assay was used to assess cell viability in response to material exposure. Fresh medium containing 0.5 mg/mL of MTT (Sigma-Aldrich, M5655, St. Louis, MO, USA) was added to the culture medium at each of the following points: 24 h, 48 h, 5 days, and 7 days. For four hours, plates were incubated at 37 °C to enable metabolically active cells to form formazan crystals. Following a cautious aspiration of the medium, the crystals were dissolved in 100 µL of dimethyl sulfoxide (DMSO). A Bio-Rad iMark microplate reader (Bio-Rad, Hercules, CA, USA) was used to measure the absorbance at 570 nm. In each independent experiment, all conditions were tested three times, and the mean absorbance of untreated control wells was used to normalize the results. Details on statistical analysis, error estimation, and propagated uncertainty for MTT assays are provided in the Supplementary Materials.

### 2.5. Gene Expression Analysis by Quantitative RT-PCR

Total RNA was extracted from MCF-7 and THP-1 cells following 24 h exposures to PETG diskettes using the RNeasy Mini Kit (Qiagen, Hilden, Germany), according to the manufacturer’s protocol. RNA concentration and purity were assessed by UV spectrophotometry (NanoDrop ND-1000, Thermo Scientific, Waltham, MA, USA), ensuring 260/280 and 260/230 ratios ≥ 1.8.

For each sample, 1 µg of total RNA was reverse transcribed using the iScript™ cDNA Synthesis Kit (Bio-Rad, Hercules, CA, USA) in a standard thermal cycler with random primers, following manufacturer instructions. Resulting cDNA was stored at −20 °C until use.

Quantitative real-time PCR (qRT-PCR) was carried out using the CFX96 Touch™ Real-Time PCR Detection System (Bio-Rad, Hercules, CA, USA), with PowerUp™ SYBR Green Master Mix (Thermo Fisher Scientific, Waltham, MA, USA). Each 20 µL reaction contained 10 µL SYBR mix, 0.5 µM of each primer, and 2 µL cDNA template. The thermal cycling protocol started by initial denaturation at 95 °C for 2 min, followed by 40 cycles of 95 °C for 15 s and 60 °C for 1 min.

Gene targets utilized for the measurements are COX-2 (PTGS2), IL-6, and IL-8 as markers of inflammatory activation in THP-1 cells. The ESR1 gene, which encodes the estrogen receptor alpha (ERα), was included to detect potential estrogenic modulation. The ESR2 gene, which encodes estrogen receptor beta (ERβ), was included as an additional marker of estrogenic modulation. ERα and Erβ thus are markers of hormonal modulation in MCF-7 cells [12]. Additional targets included IL-10, IL-1α, IL-4, TNF-α, and IFN-γ, selected to provide a broader profile of immunomodulatory responses.

Primer pairs were validated to ensure amplification efficiency between 90% and 110% and the presence of a single specific melting peak. The 2^−ΔΔCt^ method was used to determine relative expression changes, with results expressed as fold change vs. untreated controls. Glyceraldehyde-3-phosphate dehydrogenase (GADPH) was used as the endogenous control gene for normalization of expression data across all experimental conditions. Each reaction was run in technical triplicate and performed across three independent biological replicates per condition.

Details on statistical analysis, error estimation and propagation and primer’s table for qRT-PCR assays are provided in the Supplementary Materials.

## 3. Results

### 3.1. Chemical Characterization of Processed PETG Surfaces

**Figure 1:**
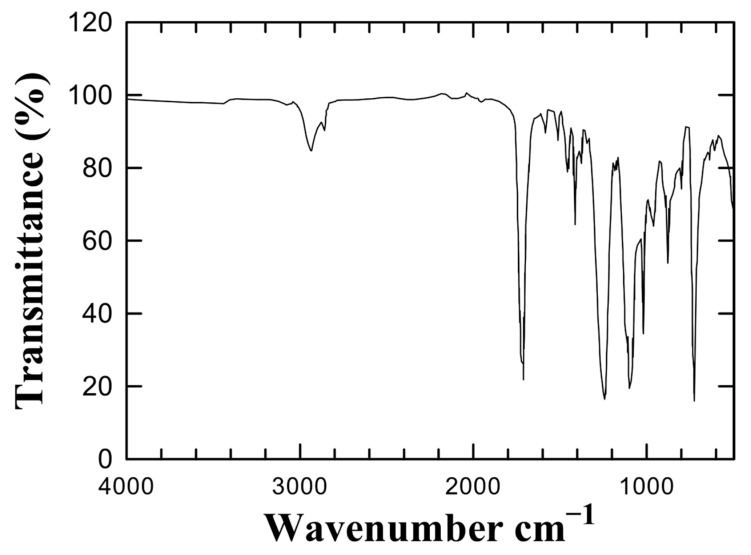
FT-IR spectrum of untreated PETG. The dominant band at ~1715 cm^−1^ corresponds to the ester carbonyl group, characteristic of polyethylene terephthalate. Secondary features near 1240 and 1100 cm^−1^ reflect C–O and aliphatic C–C stretching. No absorbance indicative of phenolic or BPA-related contaminants was detected.

Prior to any mechanical or thermal processing, chemical analyses were conducted to verify the inherent stability and inertness of the PETG polymer. Establishing this reference baseline ensured that the raw material used for disc fabrication was free from contaminants or residual monomers that could influence the biological outcomes. Untreated PETG’s FT-IR spectroscopy (Figure 1) showed spectral characteristics typical of copolymers based on terephthalates. Prominent absorptions were observed at ~1715 cm^−1^, corresponding to ester carbonyl stretching, in the range between 1504 and 1461 cm^−1^ as typical of the C-C stretching within the aromatic ring, and in the 1250–1100 cm^−1^ region, indicative of C(=O)-O stretching. The fingerprint region (600–1500 cm^−1^) showed no extraneous peaks, indicating the lack of degradation products or aromatic additives. The spectrum validated the bulk polymer’s chemical homogeneity and matched published data for medical-grade PETG materials.

**Figure 2:**
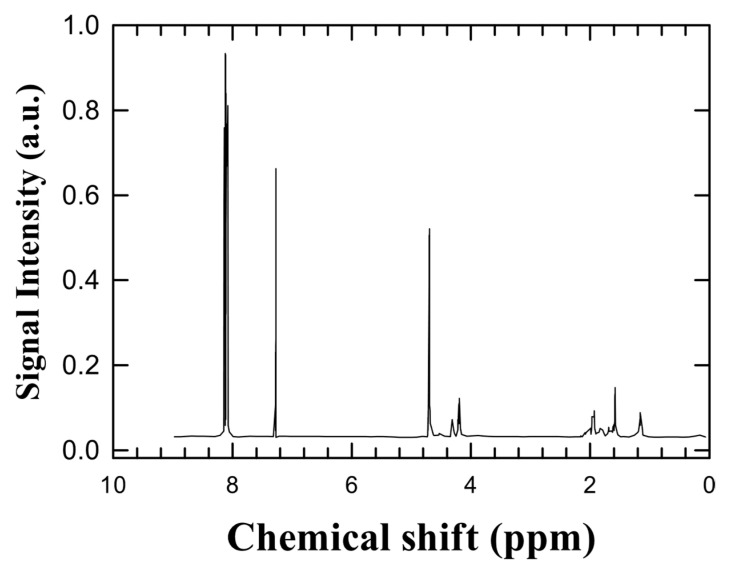
^1^H-NMR spectrum of untreated PETG. Expected aliphatic signals appeared (δ 2.0–4.5 ppm); no aromatic peaks were detected, confirming absence of BPA or phthalates.

The untreated PETG samples dissolved in DMSO-d_6_ showed the expected signals for methylene and methine protons (δ 2.0–4.5 ppm) in their ^1^H-NMR spectra (Figure 2), but the aromatic region (δ 6.5–7.5 ppm) was silent. This demonstrates that there are no free aromatic monomers, phthalates, bisphenol-A (BPA), or other hormone-disrupting substances present. These results reinforce the conclusion that the raw PETG is chemically pure and free of migratory low-molecular-weight agents.

GC-MS analysis of unprocessed PETG was performed to detect the possible presence or absence of traces of Bisphenols and any other substances. Three methanolic extracts were prepared, i.e., methanol control and two methanol sample solutions. Comparing the chromatogram of the two sample extracts with each other and with the respective Methanol White, it was observed that the signal of the dichloromethane solvent was present at about 10 min and that of the internal standard at around 28 min. After subtraction of background signals also present in the blank, no additional peaks attributable to known leachables (e.g., BPA, methacrylates, or phthalate derivatives) were observed. Collectively, these data confirm that the PETG polymer, in its untreated form, is chemically stable and free of potentially bioactive or toxic contaminants. This chemical neutrality provides a reliable baseline for subsequent biocompatibility assessments involving mechanical processing [7].

In our experiments, we intentionally adopted a bioanalytical approach that utilized two human cell lines as very sensitive detectors of surface-related effects. This functional biological readout was chosen to complement chemical analyses and to serve as a high-sensitivity screening tool capable of revealing even minimal surface alterations that may not be captured by bulk spectroscopy. Detailed biological results are presented in the following sections.

### 3.2. Cell Viability Assessment by MTT Assay

The metabolic function of MCF-7 cells grown in immediate proximity to PETG diskettes was evaluated after every 24 h, 48 h, 5 days, and 7 days with the colorimetric MTT assay. This specific assay provides an indirect but reliable measure of cell proliferation and vitality through the enzyme-catalyzed transformation of tetrazolium salts to formazan due to mitochondrial enzymes.

**Figure 3:**
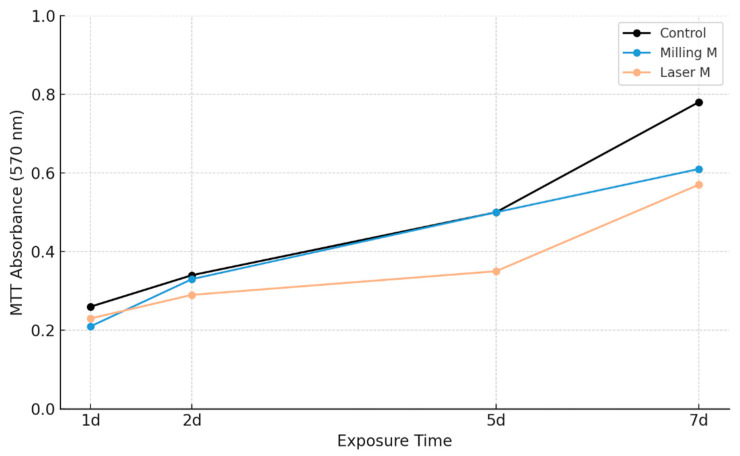
MTT assay absorbance (OD at 570 nm) measured at 1, 2, 5, and 7 days after exposure to laser- or milling-treated PETG. Data are reported as mean values. No statistically significant differences were observed among groups (one-way ANOVA, *p* > 0.05). Details on error estimation and propagated uncertainty are provided in the Supplementary Materials.

The results reported in Figure 3 show that neither laser exposure nor the milling process of PETG compromised cell viability in any of the evaluated time periods. Absorbance values for the two treated entities were slightly below that of the untreated control at the 24 h measurement point but within acceptable biological variability ranges, indicating sustained short-term viability. With the 48 h evaluation, absorbance values indicated a rise in all experimental conditions, with the absorbance values of the milled specimen equivalent to that of the control cells and signifying early proliferation in the presence of the material. By day 5, cells cultured with milled PETG showed viability values almost identical to controls, while laser-processed PETG showed slightly lower absorbance, yet still within the viable range. After 7 days of continuous exposure, MCF-7 cells maintained metabolic activity in both treatment groups, with viability exceeding 70% of the untreated control in all conditions. No evidence of growth inhibition, delayed proliferation, or cytotoxic effects was observed in any sample. The results show that PETG surfaces that are pre-processed mechanically are non-cytotoxic and release no deleterious byproducts that would adversely affect cellular viability with prolonged exposure. Slightly diminished readings in laser preparation group at subsequent assessment points are likely due to slight variations in surface roughness; however, these values are not indicative of any antiproliferative or cytotoxic activity. This finding supports the hypothesis that PETG surfaces, irrespective of the mechanical pre-treatment procedures involved, are epithelial-cell-compatible in routine vitro settings.

### 3.3. Gene Expression of Estrogen Receptors (ERα and ERβ)

**Figure 4:**
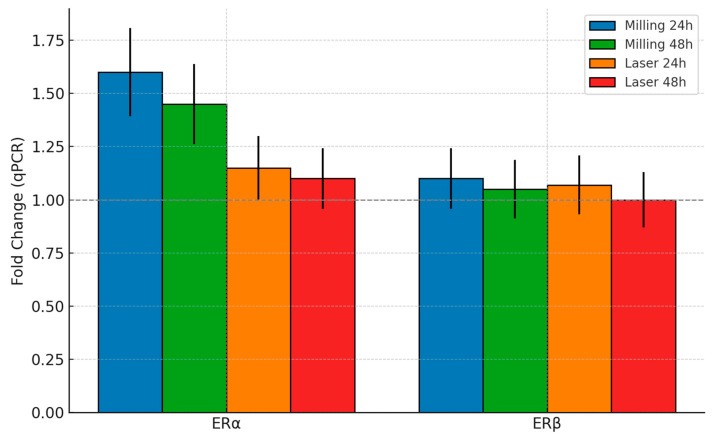
Bar graph of ERα and ERβ fold changes at 24h and 48h. Control = 1.0. Results are shown as mean values; propagated error estimates are detailed in the Supplementary Materials.

The expression levels of the *ESR1* and *ESR2* genes, which encode estrogen receptor alpha (ERα) and beta (ERβ), respectively, were measured by quantitative PCR in MCF-7 cells at 24 and 48 h to evaluate potential estrogenic effects of PETG [10] extracts. The results are shown in Figure 4, where the fold changes observed for each sample of milling-processed and laser-processed PETG during the different given time ranges are reported.

At 24 h, a slight upregulation of ERα was observed in cells treated with milling-treated PETG (1.60-fold), and a minor upregulation was observed for laser processing (1.15-fold). ERβ in both instances only showed a minor increase (1.10 and 1.05, respectively), remaining near to base levels. By the 48 h time point, ERα values displayed a minor decrease from the 24 h values but were still upregulated for the milling processing (1.45) and near to base levels for the laser processing (1.10). Across all experimental conditions and time points, ERβ expression remained stable (in the range between 1.00 and 1.05). These data indicate modest changes in ERα transcription in response to milled PETG, with no substantial variation in ERβ expression over time or treatment.

### 3.4. Gene Expression of Inflammatory Markers

**Figure 5:**
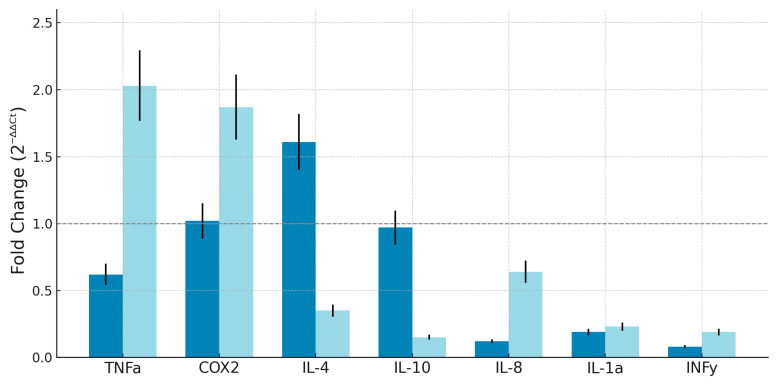
Expression of inflammatory genes in THP-1 cells after exposure to milled (left, dark bar) and laser-treated (right, light bar) PETG. Data are reported as fold changes relative to control (set to 1.0). Error treatment and propagated uncertainty are explained in the Supplementary Materials.

Figure 5 presents the relative expression levels of seven inflammatory or immunomodulatory genes (TNFα, COX2, IL-4, IL-10, IL-8, IL-1α, IFNγ) in THP-1 cultured cells in contact with PETG diskettes that were either mechanically milled or laser-processed. For each gene, expression is reported as fold change relative to untreated control cells (set to 1.0), calculated by the 2^−ΔΔCt^ method. Each gene is represented along the *x*-axis, with two adjacent bars: the lighter bar corresponds to the expression level after exposure to laser-treated PETG, while the darker bar reflects expression after exposure to milled PETG. Figure 5 enables a direct visual comparison of gene-specific transcriptional responses to the two surface processing methods. The panel includes pro-inflammatory markers (TNFα, COX2, IL-1α), regulatory cytokines (IL-10, IL-4), additional inflammatory mediators (IL-8), and a key immune signaling molecule (IFNγ). The plot illustrates the fold-change magnitude for each gene and underscores the differences in transcriptional modulation between processing treatments.

## 4. Discussion

This work is a thorough assessment of the biocompatibility of polyethylene terephthalate glycol-modified (PETG) diskettes following surface modification protocols that have clinical relevance, that is, CNC milling and CO_2_ laser cutting. By pairing judicious chemical characterizations with in vitro biological experiments on two human cell lines (MCF-7 and THP-1), the aim was to establish whether the physical modifications imposed on the surfaces of the PETG would disturb their chemical make-up or induce adverse cellular responses.

### 4.1. Chemical Stability of PETG After Post-Processing

Baseline characterization confirmed that PETG retains its bulk chemical integrity after processing, with no evidence of degradative products or toxic leachables of concern. This finding supports the conclusion that conventional shaping techniques, whether mechanical or thermal, do not compromise the material’s stability under clinically relevant conditions.

However, it is important to recognize the inherent limitations of standard chemical analyses. Techniques such as FT-IR, NMR, and GC–MS are powerful for detecting bulk degradation and soluble contaminants, yet they remain relatively insensitive to nanoscale alterations or surface-localized stresses. These subtle modifications are precisely those that may influence biological performance, especially at the cell–material interface. In this respect, cellular and molecular assays offer a higher level of sensitivity, capturing functional consequences that escape analytical chemistry but can still modulate biological outcomes.

The integration of both approaches therefore provides a more complete evaluation: chemical data confirms the absence of harmful degradation, while biomolecular assays reveal how even minimal processing-dependent changes can shape cellular responses. This dual perspective ensures that the interpretation of PETG safety is not confined to structural stability alone but also encompasses the functional relevance of surface–cell interactions.

### 4.2. Cell Viability and Metabolic Integrity

The MTT assay provided a time-resolved evaluation of cell viability and metabolic function in MCF-7 cells, chosen as a representative epithelial model with documented responsiveness to environmental stressors. This choice is particularly relevant for biomedical applications of PETG, since epithelial tissues constitute the first interface for most intraoral and mucosal devices. The assay demonstrated stable proliferation across the observation period, with viability consistently comparable to untreated controls, thereby confirming the absence of overt cytotoxicity.

The consistency of these findings is noteworthy. Maintenance of normal metabolic activity over several days suggests that neither laser finishing nor mechanical milling produced leachables or surface alterations capable of perturbing cellular energy pathways. This observation indicates that the cellular microenvironment created by the processed polymer remained compatible with healthy growth and metabolic integrity. From a regulatory standpoint, the outcome aligns with the criteria established by ISO 10993-5 [13] for in vitro cytotoxicity testing, reinforcing the conclusion that PETG, even after fabrication, can be regarded as biologically safe at the level of basal cell function.

A slight reduction in MTT absorbance was observed in the laser-treated group at later time points. This effect did not reflect toxicity but is better interpreted as a subtle response to minor changes in surface roughness or localized energy retention induced by thermal processing. Such variations show the sensitivity of the assay in detecting differences that, while biologically negligible, may still reflect physical cues at the material–cell interface. In this way, the MTT test functions not only as a safety screen but also as an indirect probe of how processing affects surface properties.

Taken together, these results confirm that processed PETG supports epithelial cell viability and preserves metabolic integrity, with no evidence of adverse effects attributable to either fabrication technique. More importantly, they underscore that biocompatibility at the level of cell viability represents only the first tier of biological assessment. The slight differences noted between processing methods, though benign, suggest that more sensitive molecular endpoints are needed to reveal whether such surface cues may exert functional influence. This provides the rationale for extending the analysis to transcriptional profiling, where early immune and endocrine markers can expose subtle but potentially relevant distinctions between milling and laser processing.

### 4.3. Estrogenic Neutrality and Endocrine Safety

The expression of estrogen receptors in MCF-7 cells represents a sensitive and biologically meaningful indicator for assessing the potential endocrine impact of processed polymers [14]. As shown in Figure 4, exposure to PETG diskettes resulted in only modest transcriptional responses, yet a clear divergence emerged between the two fabrication methods. Milling was associated with a more sustained activation of ERα, while laser finishing produced a smaller and transient effect. In both cases, ERβ expression remained essentially unchanged.

This pattern is of particular significance. Genuine estrogenic stimulation generally involves the coordinated activation of both receptor isoforms, often accompanied by downstream genes that amplify hormonal signaling [15]. The absence of ERβ modulation, combined with the lack of downstream activation, indicates that the modest ERα shifts do not reflect endocrine mimicry. Rather, they likely represent subtle adaptations to processing-induced surface features, such as microtopographic variation or localized mechanical stress, which epithelial cells are capable of sensing at the interface.

Although the magnitude of these responses was limited, the persistence of ERα activation in the milling group underscores the importance of considering long-term exposure. Clinical applications of PETG—including orthodontic aligners, retainers, and surgical guides—often involve prolonged or repeated contact with hormone-sensitive tissues [16]. Even small deviations from baseline receptor activity, when maintained over time, could theoretically contribute to low-grade signaling. In this context, the reduced and short-lived profile observed with laser finishing provides a stronger assurance of endocrine neutrality.

The biological results integrate seamlessly with the chemical analyses presented earlier. FT-IR, NMR, and GC–MS consistently excluded the presence of bisphenol A, phthalates, nonylphenol, acrylates, or related estrogenic contaminants [17], confirming the chemical stability of the polymer. The modest receptor modulation observed cannot therefore be attributed to leachables with hormonal activity but must instead arise from surface-level phenomena intrinsic to the processing method. This convergence between chemical and biological data strengthens the conclusion that PETG, under the tested conditions, does not generate biologically relevant endocrine-disrupting species.

In summary, the endocrine assays confirm that PETG retains hormonal safety following both laser and milling treatments. Milling induces a mild but sustained ERα response without ERβ co-activation, whereas laser finishing results in negligible and transient changes. Both outcomes fall within ranges considered biologically acceptable, but laser treatment appears to offer a more conservative safeguard in clinical scenarios where strict endocrine neutrality is required.

### 4.4. Inflammatory Gene Expression: A Marker of Subtle Cellular Stress

The transcriptional behavior of inflammatory mediators in THP-1 monocytes after exposure to processed PETG surfaces provides valuable insight into how subtle changes in surface properties can shape immune signaling. [18]. As shown in Figure 5, the expression of a representative panel of cytokines was profiled, including classical pro-inflammatory markers such as TNFα, COX-2, IL-1α, IL-8, and IFNγ, together with regulatory mediators IL-4 and IL-10. Across this spectrum, responses were modest, generally remaining within the range conventionally regarded as biologically acceptable in biomaterials testing. The overall impression is one of stability: neither milling nor laser finishing triggered a broad inflammatory cascade [19].

Closer inspection, however, reveals interesting contrasts between the two processing modalities. Milling consistently produced an attenuated inflammatory profile, characterized by reduced or unchanged levels of major pro-inflammatory mediators and modest increases in regulatory cytokines. This suggests that mechanical modification, while altering surface topography, does not stimulate immune activation and may instead favor a more balanced or tolerogenic response [20,21]. By contrast, laser finishing was associated with enhanced transcription of early stress-responsive markers, including TNFα and COX-2, often accompanied by a parallel though moderate increase in IL-8. Yet this activation did not propagate into systemic pathways: both IL-1α and IFNγ remained close to baseline, confirming that the response was restricted to local signaling and did not escalate into a full immune program.

The divergent regulation of IL-4 and IL-10 further underscores this difference. Milling promoted modest elevations of these immunoregulatory cytokines, suggesting a tendency toward compensatory or anti-inflammatory signaling. Laser treatment, conversely, was accompanied by their suppression, consistent with a shift toward a more neutral-to-pro-inflammatory equilibrium. Taken together, these patterns indicate that the two processing strategies elicit distinct transcriptional fingerprints: milling biases the response toward regulation, whereas laser finishing transiently tips the balance toward activation.

Importantly, none of these alterations exceeded thresholds associated with toxicological concern, and several trends lacked statistical robustness or reproducibility across replicates. These findings argue against any meaningful immune stimulation under the short-term exposure conditions tested. Nevertheless, the possibility that even subtle, low-grade changes may gain relevance under chronic or repeated exposure cannot be dismissed. In particular, a surface that consistently promotes pro-inflammatory cues, even if moderate, might contribute to mucosal stress during long-term intraoral application, whereas a surface that enhances regulatory signaling could provide a more favorable immunological environment.

Overall, the transcriptional assays reinforce the chemical evidence for the biological safety of PETG, but they also highlight the added value of molecular profiling. Standard bulk analyses such as FT-IR, NMR, and GC–MS confirm chemical stability but are inherently less sensitive to nanoscale or surface-localized modifications. Cellular assays, by contrast, can capture these subtle differences, offering a functional readout of biological impact that might otherwise remain undetected. In this respect, the combined approach adopted in this study provides a comprehensive framework for assessing the biological neutrality of processed PETG, while also identifying context-dependent variations that could influence its performance in clinical settings.

### 4.5. Cross-Validation in Epithelial and Monocytic Models

An advantage of the present study is the inclusion of two human-derived cell lines with distinct physiological roles. MCF-7 cells represent epithelial interfaces (Figure 4), relevant for most tissue–material interactions [22], while THP-1 monocytic cells provide a conservative model for immunological sensitivity [23,24]. By testing both, we were able to assess whether the observed molecular responses reflect lineage-specific effects or more general cell–surface interactions.

The responses observed in THP-1 cells (Figure 5) reinforce the conclusion that PETG, even when processed mechanically, does not trigger a classical inflammatory cascade. After exposure to milling procedure, only minimal COX-2 upregulation was observed, while pro-inflammatory mediators such as IL-8 were reduced. Laser-processed PETG caused an enhanced transcription activity for COX-2, but this upregulation took place without a corresponding upregulation of IL-1α or IFNγ and so confirming the absence of more global immune activation. The gene modulation observed therefore reflects localized cellular sensing of stress rather than systemic or adverse immune activation.

When interpreted alongside the epithelial model, these findings support the primary aim of the study: to evaluate the chemical stability and biological neutrality of PETG under clinically relevant processing conditions. The absence of cytotoxicity, sustained metabolic activity, and only modest transcriptional shifts in both cell lines suggest that surface alterations induced by milling or laser finishing do not result in biologically significant consequences. While the study relied on biological endpoints as indirect indicators of surface changes, future work could incorporate direct physical analyses (e.g., SEM imaging or contact angle measurements) to complement and extend these observations.

### 4.6. Comparative Performance: Laser vs. Milling

Both laser ablation and CNC milling are commonly employed techniques for shaping PETG in biomedical applications. Importantly, both approaches preserve the bulk chemical integrity of the polymer, which underpins its stability and native biocompatibility [2]. Yet, comparative cellular and molecular evaluations revealed distinct fingerprints of biological interaction that highlight subtle but meaningful differences between the two processing methods.

MTT viability assays demonstrated that both treatments supported sustained and healthy proliferation of MCF-7 cells. A consistent though modest elevation of metabolic activity was observed in the laser-treated group compared with milling. While this difference did not reach statistical significance, it may indicate reduced strain on cells or improved compatibility with the laser-finished interface. In either case, the data confirm that both techniques avoid cytotoxic effects but suggests that surface-related factors such as microtopography or energy retention can influence the quality of epithelial cell–material interactions.

Gene expression analyses in THP-1 monocytes provided further insight into these contrasts [23]. Laser processing induced stronger activation of early pro-inflammatory genes, including TNFα and COX-2, with a modest but measurable increase in IL-8 as well. COX-2, a key indicator of primary inflammatory and stress responses, showed the highest expression under laser treatment, pointing to a mild stress-related signature. Milling, in contrast, resulted in reduced expression of TNFα and IL-8, alongside an elevation of the regulatory cytokines IL-4 and IL-10. These findings suggest that milling promotes a more balanced or compensatory immunoregulatory response, while laser finishing tends toward transient pro-inflammatory activation. Notably, IL-1α and IFNγ remained stable in both groups, and the magnitude of all responses fell within the conventional ±2-fold variation considered acceptable in biomaterials testing, underscoring the absence of systemic or clinically significant immune activation.

Parallel differences emerged in hormone-responsive MCF-7 cells. ERα (ESR1) exhibited more persistent induction following milling, whereas laser-treated PETG surfaces generated only a smaller and transient response. ERβ (ESR2) remained unchanged in all conditions, and the absence of coordinated receptor modulation or downstream estrogen-responsive genes argues strongly against genuine endocrine disruption. Taken together, these findings indicate that laser finishing better preserves endocrine neutrality, a consideration of particular importance for applications in hormone-sensitive anatomical regions.

In summary, both laser and milling treatments result in chemical stability. In summary, both laser ablation and mechanical milling yield chemically stable and biologically safe PETG surfaces. However, their biological signatures diverge: milling tends to support a more immunomodulatory balance, while laser finishing better conserves endocrine neutrality. These context-specific outcomes reinforce the importance of aligning processing strategies with clinical priorities: milling may be advantageous in immune-sensitive settings, whereas laser treatment could be preferred in applications where strict hormonal neutrality must be ensured.

### 4.7. Clinical and Regulatory Relevance

These findings have direct implications for clinical applications of PETG, which are increasingly used in the fabrication of patient-specific surgical guides, orthodontic aligners, and drug delivery platforms—devices that require extensive and direct contact with human tissues. The results of this study indicate that established processing techniques, namely laser and mechanical surface modifications, introduce no biologically concerning molecular alterations that might pose safety concerns in clinical practice. The lack of notable immunological or hormonal gene activation indicates the material’s suitability for sensitive biological contexts. We acknowledge that the use of immortalized cell lines, while reproducible and mechanistically informative, does not fully recapitulate the complexity of oral mucosal tissues. Future studies using primary gingival fibroblasts or oral keratinocytes will be necessary to confirm the long-term intraoral biocompatibility of processed PETG. However, the combined use of two mechanistically distinct human cell lines in this study—monocytic and estrogen-responsive—offers a robust and sensitive platform for first-level screening of functional material effects.

The combined chemical and biological evaluation approach outlined here complies with ISO 10993-5 requirements for in vitro cytotoxicity testing, supporting the biological safety of PETG after processing. Moreover, this integrated preclinical pipeline may be adapted to strengthen regulatory submissions under frameworks such as REACH (for chemical safety) and FDA 510(k) premarket notification, particularly for Class II medical devices involving prolonged mucosal or dermal contact [25].

Future studies using primary gingival fibroblasts or oral keratinocytes will be necessary to confirm the long-term intraoral biocompatibility of processed PETG. It should also be noted that this study does not replicate key oral environmental factors such as salivary enzymes, microbial flora, mechanical stress, or thermal cycling, which may influence the long-term behavior of materials during intraoral use. These limitations should be considered when interpreting the findings, which are intended as an initial biological screening under controlled in vitro conditions.

## 5. Conclusions

This study provides robust evidence that polyethylene terephthalate glycol-modified (PETG), a widely used thermoplastic in biomedical device manufacturing, retains its chemical stability and biological safety following surface processing by CNC milling or CO_2_ laser cutting.

Chemical investigations confirmed the absence of degradation products, while in vitro assays using MCF-7 and THP-1 human cell lines showed no evidence of cytotoxicity, inflammatory activation, or endocrine disruption under simulated physiological conditions.

Overall, PETG processed with standard clinical techniques can be considered safe from both chemical and cellular standpoints. These results support its continued use in patient-specific medical devices and suggest that the integrated evaluation strategy applied here may serve as a replicable model for the preclinical validation of other biomedical materials. Future studies using primary gingival fibroblasts or oral keratinocytes and exploring physical surface analyses (e.g., SEM or contact angle measurements) are needed to complement and expand the current findings.

## Data Availability

The original contributions presented in this study are included in the article/Supplementary Material. Further inquiries can be directed to the corresponding author alessandro.ugolini@unige.it.

## Supplementary Materials

The following supporting information can be downloaded at: https://www.mdpi.com/article/10.3390/ma18204793/s1.

## Abbreviations

The following abbreviations are used in this manuscript:

PETG	Polyethylene terephthalate glycol-modified
CNC	Computer Numerical Control
FT-IR	Fourier Transform Infrared
^1^H-NMR	Proton Nuclear Magnetic Resonance
GC-MS	Gas Chromatography–Mass Spectrometry
MCF-7	Michigan Cancer Foundation-7
THP-1	Tohoku Hospital Pediatrics-1
MTT	3-(4,5-dimethylthiazol-2-yl)-2,5-diphenyltetrazolium bromide.
COX-2	Cyclooxygenase-
TNFα	Tumor Necrosis Factor alpha
IL	Interleukin
IFNγ	Interferon-gamma
ER	Estrogen Receptor
ATR	Attenuated total reflectance
ATCC	American Type Culture Collectio
DMEM	Dulbecco’s Modified Eagle Medium
PBS	Phosphate-Buffered Saline
DMSO	dimethyl sulfoxide
GADPH	Glyceraldehyde-3-phosphate dehydrogenase
FBS	Fetal Bovine Serum
BPA	Bisphenol A
