# Supplementary material for: Molecular Biocompatibility Assessment of PETG Aligners After Processing by Laser or Milling
Source: Materials (Basel). 2025 Oct 20;18(20):4793. doi: 10.3390/ma18204793 (PMC12566054; doi:10.3390/ma18204793)
Supplement: Supplementary file 1 [file materials-18-04793-s001.zip › materials-3906263-supplementary.pdf]

Supplementary materials

Molecular Biocompatibility Assessment of PETG Aligners after Processing by Laser or Milling

SUPPLEMENTARY MATERIAL

Treatment and Representation of Experimental Errors

In this study, three major data domains were evaluated—chemical characterization, cell viability, and gene expression—each associated with distinct sources of error. These were addressed using a combination of statistical and instrument-based approaches, with additional conservative assumptions when appropriate.

For chemical analyses such as FT-IR spectroscopy, proton NMR, and GC-MS, the precision of modern instruments results in extremely low measurement variability. These techniques were used to confirm material identity and chemical stability, not to quantify subtle changes. As such, error estimation was not required for graphical representation; the outcome was qualitative in nature and focused on the presence or absence of new or altered spectral features.

In contrast, metabolic activity measured by the MTT assay involves several layers of uncertainty, including biological variability, pipetting error, and instrumental noise from spectrophotometric readings. When multiple replicates were available, standard deviations were calculated and analyzed via one-way ANOVA to test for statistically significant differences across treatment groups. However, in cases where replication was limited or variance was extremely low, we recognized the risk of underrepresenting biological variability. For this reason, a conservative minimal error band of approximately ±10% was used for visual clarity in the representation of cell viability data when standard deviation fell below this threshold.

Gene expression analysis using qRT-PCR presents a more complex case. The raw measurement is the Ct value—the cycle at which fluorescence crosses a threshold. These Ct values are then normalized against housekeeping genes and compared across experimental groups using the ΔΔCt method. The final biological output is reported as fold change (2^–ΔΔCt), which is a nonlinear transformation. When ΔΔCt is accompanied by replicates, the standard deviation can be propagated using established formulas for exponential functions. Specifically, the propagated error in fold change ($y = 2^{-x}$) is given by:

$$\delta y = \ln(2) \cdot 2^{-x} \cdot \delta x$$

Using this relationship, a typical ΔΔCt standard deviation of ±0.3 translates to a fold change uncertainty of approximately ±13%, depending on the value of x. For example, if ΔΔCt = 1 (i.e., fold change = 0.5), the propagated error is 0.5 × ln(2) × 0.3 ≈ ±0.104, or 20.8%. However, for ΔΔCt = 0.3 (fold change ≈ 0.81), the error becomes ≈ ±0.081, or ~10%. Across the range of fold changes observed in this study, the average propagated uncertainty stabilizes around 13%, which we adopted as the standard error band for graphical representation of gene expression.

This 13% error was used consistently when replicates were limited or when observed standard deviations were unrealistically small, which can occur in high-efficiency qPCR instruments due to minimal cycle variability. While this estimate does not replace formal statistical testing, it serves as a realistic and evidence-based approximation of overall methodological uncertainty, including variability from reverse transcription, primer efficiency, and sample handling.

Wherever sufficient replicates were available (n ≥ 3), statistical comparisons between groups were carried out using one-way ANOVA followed by post hoc testing. These were used to support conclusions regarding differences in gene expression but were not always indicated in figures to preserve clarity when showing multi-gene comparisons across treatments.

Overall, the combined strategy employed in this study—using ANOVA-based statistics when available and a consistent ±13% propagated error otherwise—allowed for the accurate and responsible interpretation of results across multiple assays. This approach ensures that conclusions about the biocompatibility and biological neutrality of PETG are supported by both robust statistical evidence and a cautious yet realistic treatment of measurement uncertainty.

**Gene**	**Target**	**Forward Primer (5→3)**	**Reverse Primer (5→3)**	**Amplicon Size (bp)**	**Annealing Temp (°C)**
**COX-2 (PTGS2)**	Inflammation	GTTCCACCCGCAGTACAGAA	AGGGCTTCAGCATAAAGCGT	106	58
**IL-6**	Pro-inflammatory	AGACAAAGCCACCACCCCTAA	CTCGTTCTGTGACTGCAGCTTATC	120	60
**IL-8**	Chemokine	GAAAACTGGGTGCAGAGGGT	AAAAAGGCAGATACCTAATGACGA	156	57
**IL-4**	Anti-inflammatory	CCAACCCTGGTCTGCTTACTG	TTGTAAGGTGATGTCGCACTTGT	140	58
**IL-10**	Anti-inflammatory	GCCTTCGGCCCAGTGAA	AGAGACCCGGTCAGCAACAA	346	54

**TNF-α**	Pro-inflammatory	AAAACAACCCTCAGACGCCA	CATGGTGTCCTTTCCAGGGG	108	58
**IFN-γ**	Pro-inflammatory	TGTCGCCAGCAGCTAAAACA	ACTGGGATGCTCTTCGACCT	72	58
**ERα (ESR1)**	Hormonal	CACATGAGTAACAAAGGCATGG	ATGAAGTAGAGCCCGCAGTG	177	56
**ERβ (ESR2)**	Hormonal	GGCATGCGAGTAACAAGGGC	GGGAGCCCTCTTTGCTTTT	177	56
**GAPDH**	Housekeeping	ACCAGGGCTGCTTTTAAC	ACGGTGCCATGGAATTTG	125	54
